# Reduction of elevated lipids and low‐density lipoprotein oxidation in serum of individuals with subclinical hypoxia and oxidative stress supplemented with lycosome formulation of docosahexaenoic acid

**DOI:** 10.1002/fsn3.784

**Published:** 2019-03-20

**Authors:** Ivan M. Petyaev, Pavel Y. Dovgalevsky, Natalia E. Chalyk, Victor A. Klochkov, Nigel H. Kyle

**Affiliations:** ^1^ Lycotec Ltd Cambridge UK; ^2^ Institute of Cardiology Saratov Russia

**Keywords:** docosahexaenoic acid, hyperlipidemia, LDL oxidation, Lycopene, triglyceride and LDL reduction

## Abstract

Thirty two individuals aged 40–65 years old with a moderate hyperlipidemia (serum triglycerides > 150 mg/dl and LDL from 130 to 160 mg/dl) were supplemented once daily for 30 days with a 250 mg conventional formulation of docosahexaenoic acid (DHA) without lycopene (CF‐DHA) or 250 mg of lycosome‐formulated DHA containing 7 mg of lycopene (LF‐DHA). It was shown that ingestion of CF‐DHA led to a transient increase in serum DHA level after 2 weeks of the trial, whereas LF‐DHA did not cause significant changes in serum DHA. However, there was a noticeable increase in serum eicosapentaenoic acid levels exceeding the pretreatment value by 42.8% and 39.1% after the 2nd and 4th weeks of LF‐DHA ingestion. Patients supplemented with LF‐DHA showed a significant (19.5 mg/dl, *p* < 0.05) decline in LDL, which was accompanied by a corresponding decrease in total serum cholesterol and a much stronger reduction in serum triglyceride levels (reduction of medians by 27.5 mg/dl). No changes in HDL were observed. LF‐DHA caused a significant decline in the serum level of malonic dialdehyde (MDA), whereas the components of LF‐DHA, lycopene and DHA, ingested as two separate formulations had a less significant effect on serum MDA. Moreover, LF‐DHA increased both the plasma oxygen transport and tissue oxygen saturation by the end of the observational period, while lycopene or DHA taken alone, or both of them co‐ingested separately had none or a much less effect on the oxygen turnover parameters.

## 1 INTRODUCTION

The epidemiological link between marine‐derived omega‐3 polyunsaturated fatty acids (n‐3‐PUFAs) and incidence of cardiovascular disease (CVD) originates from classic analysis of the health status of the Greenlandic Eskimo population which is characterized by high consumption of fatty fish, low cardiovascular mortality, nonatherogenic lipid profile, reduced platelet counts, longer blood clotting times, and raised concentration of PUFA in platelet membranes (Bowen, Harris, & Kris‐Etherton, 2016; Maehre, Jensen, Elvevoll, & Eilertsen, 2015). During the past 40 years, high n‐3‐PUFA consumption, either in the form of dietary intake of fatty fish or as docosahexaenoic acid (DHA)/eicosapentaenoic acid (EPA) supplements, has been shown in experimental and clinical settings to have favorable dose‐dependent effects on the parameters of various systems—cardiovascular, neurological and reproductive as well as on intermediate metabolism (Belkouch et al., 2016; Bowen et al., 2016; Jackson & Harris, 2016; Maehre et al., 2015).

Polyunsaturated fatty acids have a highly distinctive chemical structure epitomized by a hydrocarbon chain with a carboxyl group and a methyl group at opposing ends with a double bond at the third carbon atom. Hydrophobicity is a key feature of PUFA molecules and a major factor limiting PUFA bioavailability. Intestinal absorption of PUFA requires vigorous emulsification and depends on bile acid as well pancreatic lipase secretion rates into the intestinal lumen (Ghasemifard, Hermon, Turchini, & Sinclair, 2015; Ulven & Holven, 2015). Various nutraceutical formulations of DHA/EPA with improved bioavailability to allow reduced daily DHA intake and minimize possible DHA side effects have been proposed recently (Ghorbanzadze, Jafari, Akhavan, & Hadavi, 2017; Puri et al., 2016).

In the present study, we report pharmacokinetics and pharmacodynamics data for a new formulation of DHA which was developed based on lycosome delivery technology (Petyaev, 2012, 2016a,2016b). The key ingredient of this delivery vehicle is lycopene, a carotenoid which has particular affinity for the liver; hence, it can serve as a vector for hepatocyte targeted delivery. Lycopene also provides a significant level of protection for “cargo” molecules, such as DHA, from stomach acidity and oxidation by digestive enzymes. In a water‐free environment, lycosome suspension is composed of microparticles with an enhanced intestinal absorption rate is capable of penetrating the gut in a partially unmodified form. Previously, lycosome technology was successfully used (Bashmakov et al., 2014; Petyaev, 2012; Petyaev, Dovgalevsky, Chalyk, Klochkov, & Kyle, 2014; Petyaev, Dovgalevsky, Klochkov, Chalyk, & Kyle, 2012) to increase the bioavailability of different nutraceuticals (whey protein peptides, cocoa polyphenols, and resveratrol) and pharmaceuticals (HMG‐CoA reductase inhibitors).

As shown below, lycosome formulation of DHA not only has an enhanced its triglyceride‐lowering efficacy but added new modalities: LDL‐lowering, boost of peripheral tissue oxygenation and reduction of markers of oxidative damage in middle aged individuals after one month of treatment.

## 2 MATERIALS AND METHODS

### 2.1 Study design

The study was conducted at the Institute of Cardiology, the Ministry of Health of the Russian Federation (Saratov, RF). The trial was part of a broader nutraceutical trial registered (ACTRN12613000966796) and approved by the local ethics committee. All patients were fully informed about the purpose of the study and gave written consent for their participation in the clinical study.

### 2.2 Inclusion/exclusion criteria

This study was part of a larger multi‐arm trial which included 89 patients with not only elevated serum triglyceride and cholesterol but also with markers of subclinical hypoxia and oxidative damage who were not taking any medication or supplements affecting oxygen turnover and oxidative status. Oxidative stress was defined as a status characterized by increased level of oxidized LDL and upregulated values for malonic dialdehyde in serum. Subclinical hypoxia was defined as a state with abnormally low values for plasma oxygen transport parameters and lowered values for tissue oxygen saturation—StO_2_. Patients positive for both groups of markers (hypoxia and oxidative stress) were recruited for the study and underwent thorough clinical and anamnestic investigation. Two weeks later, all patients were rescreened and randomized into four study groups (eight patients per group), and given 2 weeks supply of study products. All tests were carried out at the baseline and intermediate time point, 14 days after intake of the investigational products, and at completion of the study after 28 days. Patients were given a further 2 weeks supply of the study products at an intermediate visit. Clinical tests and examinations were carried out at the beginning of the study and at its end. Three patients were unable to complete the study for various reasons not related to the intake of the test products and were replaced with qualifying individuals from the prescreened pool. Patient illegibility was determined by applying inclusion/exclusion criteria.

#### 2.2.1 Inclusion criteria

Caucasian male or female subjects 40–65 years with: serum triglyceride concentration > 150 mg/dL and total cholesterol > 200 mg/dL with signed consent form; no anti‐hypertensive, lipid‐lowering, or anti‐diabetic drugs; serum‐positivity for markers of inflammation and oxidation such as oxidized low‐density lipoprotein (LDL‐Px ELISA) ≥200 U; Inflammatory Oxidative Damage (IOD) values ≥40 μM of malonic dialdehyde as well as willingness and ability to comply with the protocol for the duration of the study; abnormal values of oxygen turnover which include tissue oxygen saturation—StO_2_ < 90.0 StO_2_ (% O_2_/min) and plasma oxygen transport – <700.0.

#### 2.2.2 Exclusion criteria

Unwillingness to sign the informed consent, inability to comply with the protocol of the study, significant medical condition that would impact safety considerations (severe CVD, hepatitis, severe dermatitis, uncontrolled diabetes, cancer, severe GI disease, fibromyalgia, renal failure, recent cerebrovascular accident, pancreatitis, respiratory diseases, epilepsy, HIV/AIDS) and compulsive alcohol abuse (*>*10 drinks weekly), or regular exposure to other substances of abuse, concurrent participation in other dietary studies or clinical studies, allergy to omega‐3 supplements or fish oil.

Individual dietary patterns were assessed and recorded during the enrolment process and used for the randomization procedure. All participants were asked to adhere to a habitual dietary pattern during the study. No adverse effects were reported during the study.

### 2.3 Study products

#### 2.3.1 Conventional formulation of DHA

Individuals from the first group took daily one capsule containing 250 mg of DHA (DSM, Switzerland).

#### 2.3.2 Lycopene

Volunteers from the second group were asked to take daily one capsule of 7 mg of lycopene (Lycored, Switzerland).

#### 2.3.3 Co‐ingestion of DHA and lycopene

Two separate capsules of 250 mg of DHA and 7 mg of lycopene were simultaneously ingested by volunteers in the third group of the study.

#### 2.3.4 Lycosome formulation of DHA

Individuals from the fourth group were instructed to ingest daily one capsule containing 250 mg of lycosome‐formulated DHA containing 7 mg of lycopene (Lycotec, UK). As has been shown in our previous work, lycosome delivery technology and the presence of lycopene in lycosome‐formulated products improves bioavailability of certain nutraceuticals by increasing their resistance to oxidation in the gastrointestinal lumen, promoting their intestinal absorption rate and hepatic delivery (Bashmakov et al., 2014; Petyaev, 2012; Petyaev et al., 2012, 2014).

All study products were ingested once daily for 4 weeks (28 subsequent days) in the morning with breakfast meals.

### 2.4 Blood collection, biochemistry, and inflammatory markers

Blood was collected in the morning between 8 a.m. and 10 a.m. following night fast, from arm veins of the patients. The serum was separated by centrifugation and aliquots stored at −80°C prior to analysis. Glucose, total cholesterol, TC, triglycerides, TG, high‐density cholesterol, HDL, low‐density cholesterol, LDL, C‐reactive protein, CRP were measured using commercially available analytical kits according to the manufacturers’ instructions (ByoSystems, R&D Systems).

#### 2.4.1 Lycopene quantitative analysis

The lycopene concentration in all serum samples was measured in duplicate by high‐performance liquid chromatography (Diwadkar‐Navsariwala et al., 2003) with modifications. Briefly, 400 μl of serum was mixed with 400 μl of ethanol and was extracted twice with 2 ml hexane. The combined hexane layers were evaporated to dryness in a vacuum (Scan Speed 32 centrifuge) and the residue reconstituted to a volume of 100 μl in sample solution (absolute ethanol – methylene chloride, 5:1, v/v). The specimens were centrifuged again (15 min at 10,000 g), and clear supernatant was transferred to HPLC vials. Five microliters of the extract was injected into an Acquity HSS T3 75 × 2.1 mm 1.8 μm column (Waters, USA) preceded by an Acquity HSS T3 1.8 μm VanGuard precolumn (Waters) and eluted isocratically at 45°C with the mobile phase (acetonitrile—0.08% phosphoric acid solution—tert‐butyl methyl ether, 70:5:25, v/v/v) at a flow rate of 0.5 ml/min. The lycopene peak was detected by a Photodiode Array Detector (Waters) at 474 nm. The peak area was measured using Empower 3 software (Waters, MA). The lycopene concentration in serum samples was calculated by reference to an analytical standard (lycopene from tomato, L‐9879; Sigma, USA).

### 2.5 Serum DHA and EPA measurments

EPA and DHA concentration in all serum samples was measured in duplicate by gas‐liquid chromatography (Bowen, Kehler, & Evans, 2010), with slight modifications. Briefly, the 2 ml of stock solution required for each sample included 1.9 ml of methanol and 100 μl of acetyl chloride. Briefly, 100 μl of serum and 2 ml of the stock solution were combined in screw‐capped glass tubes. The tubes were capped and heated at 100°C for 60 min. The tubes were allowed to cool down to room temperature and were extracted twice with 1 ml hexane. The combined hexane solution was evaporated under vacuum (Scan Speed 32 centrifuge) and the residue reconstituted to a volume of 50 µl with hexane, transferred to GC vials, and capped under nitrogen.

Fatty acid analysis was performed with a fused silica capillary column (HP‐5), 30 m 0.32 mm inner diameter (ID), 0.25 m film thickness (Hewlett Packard, USA), a Shimadzu GC 2010 Gas chromatograph with Flame Ionization Detector and manual injection system (Shimadzu, Japan). Temperature program, initial: 130°C with a 4‐min hold; ramp: 4°C/min to 280°C with a 2‐min hold. Carrier gas was He, with a linear velocity of 30 cm/s. Fatty acid analysis was performed by injection of 1 μl of each sample at a split ratio of 50:1. The FID and the Injection port temperature was 300°C. The sampling frequency was 40 Hz. Fatty acid identification was performed with SUPELCO 37 COMPONENT FAME MIX (Supelco, USA). DHA and EPA concentrations in serum samples were calculated on the basis of standard concentrations. Analytical Standards (EPA methyl ester and docosahexanoic acid; Sigma, USA) were used for the assay.

### 2.6 Tissue oxygen saturation

As a tissue target for the assessment of oxygen saturation, StO_2_, or combined level of oxygenated hemoglobin and myoglobin, we used the thenar eminence and forearm muscles of the patients (Comerota, Throm, Kelly, & Jaff, 2003). StO_2_ was analyzed by continuous wavelength near‐infrared spectroscopy, NIRS, with wide‐gap second‐derivative (In Spectra, Hutchinson Technology, MN, USA). The measurements were made at different time‐points. The recording began following 15‐min rest in a supine position before occlusion of the brachial artery. It then continued during stagnant ischemia induced by rapidly inflating the cuff to 50 mmHg above systolic BP. The ischemia lasted for 3 min, and the recording period lasted for another 5 min after that until StO_2_ was stabilized.

Then, the area under the hyperemic curve, AUC, of the recorded signal for the settling time in the postocclusion period was calculated as described previuosly in % O_2_/minute.

### 2.7 Plasma oxygen transport

Plasma oxygen transport parameters were measured as described elsewhere (Lin et al., 2010).

### 2.8 Statistics

For the assessment of normally distributed parameters, the Shapiro‐Wilk method was used. Student's * t* test was then applied both for paired and unpaired samples. In cases where parameters were not normally distributed the Mann‐Whitney U‐test and Kruskal‐Wallis test were used. ANOVA and ANCOVA were used with post hoc analysis (Statistica 9 suite, StatSoft Inc.). Statistical significance between two‐tailed parameters was considered to be *p* < 0.05.

## 3 RESULTS

### 3.1 Baseline values

Table 1 shows that all baseline parameters for the patients enrolled in the study were similar, and that there were no significant differences in gender, age or body mass index among the four major groups of the study. Moreover, serum glucose and lipids (total cholesterol, triglycerides, HDL, and LDL) were similar among all individuals. It should also be emphasized that individuals belonging to the four major groups of the study showed no differences in serum DHA and EPA levels. It was also evident that habitual dietary regimen with no superfluous DHA supplementation provided an amount of omega‐3 fatty acids sufficient to maintain serum DHA and EPA concentrations at detectable and measurable values.

**Table 1:** Baseline characteristics of the enrolled patients (averages ± standard deviations)

Variable	DHA alone	Lycopene alone	Co‐ingestion DHA and lycopene	Lycosome formulation DHA and lycopene
Number of patients	8	8	8	8
Males	4	4	4	4
Females	4	4	4	4
Age	54.62 ± 4.27	57.50 ± 2.59	57.37 ± 2.54	58.87 ± 2.71
Light/moderate smokers	1/8	2/8	1/8	1/8
Body Mass Index	22.18 ± 0.98	22.57 ± 0.61	22.63 ± 0.69	22.38 ± 0.43
Fasting glucose mg/dl	86.75 ± 5.42	88.12 ± 3.05	86.31 ± 6.08	84.25 ± 5.47
Total cholesterol mg/dl	223.00 ± 14.72	227.00 ± 13.38	223.5 ± 9.75	231.75 ± 7.79
Triglycerides mg/dl	194.002 ± 10.34	202.12 ± 6.95	197.37 ± 7.24	193.87.3 ± 8.72
LDL mg/dl	154.00.1 ± 3.03	153.37 ± 3.73	155.50 ± 4.78	153.32.3 ± 3.23
HDL mg/dl	41.4 ± 2.3	43.9 ± 1.94	42.5 ± 2.4	41.1 ± 2.3
Plasma oxygen transport	635.25 ± 11.68	649.87 ± 4.80	641.62.3 ± 11.89	640.25 ± 7.42
StO_2_	72.75 ± 2.53	75.50 ± 1.93	75.00 ± 2.44	75.25 ± 1.71
Serum DHA	89.62 ± 9.04	90.75 ± 8.66	90.87 ± 7.13	85.12 ± 8.68
Serum EPA	42.12 ± 4.75	43.50 ± 3.42	45.62 ± 3.38	49.25 ± 3.26

Notes

The volunteers were screened, enrolled, and randomized into the four major groups of the study as described in the “Materials and Methods” section. Baseline parameters were measured at the “0” time‐point of the trial and shown above.

### 3.2 DHA, EPA, and lycopene

Table 2 summarizes the results reflecting serum levels of DHA and EPA for the individuals ingesting different formulations of DHA and lycopene over the course of the study. As can be seen, ingestion of the regular formulation of DHA lead to a small but statistically significant increased serum DHA level after 2 weeks of consumption (increase of medians by 23.0 μg/ml, *p* < 0.05), whereas a 4‐week ingestion of DHA was accompanied by a much less significant increase in serum DHA falling below the point of statistical significance (*p* > 0.05). As expected, ingestion of lycopene alone did not affect serum DHA levels. Interestingly, co‐ingestion of lycopene and DHA taken as two separate formulations did reproduce exactly the same quantitative pattern of serum DHA changes over the course of the study. As can be seen from Table 2, an initial increase in serum DHA after 2 weeks of supplementation had shifted to normal values for DHA in serum at the end‐point of the study. Therefore, mere co‐ingestion of DHA and lycopene as two separate formulations does not affect serum level of DHA. Interestingly, ingestion of the lycosome formulation of DHA did not change the serum level of DHA at all.

**Table 2:** Serum DHA and EPA levels (medians with 95%/5% Cis)

Time point	DHA μg/ml				EPA μg/ml			
	DHA	Lycopene	Co‐Ingestion	Lycopene‐DHA	DHA	Lycopene	Co‐Ingestion	Lycopene‐DHA
“0” week	92.5 75.0/98.3	92.0 85.7/95.1	90.5 80.7/99.9	83.5 73.7/96.3	43.0 35.7/48.9	43.5 38.4/48.0	45.5 41.3/50.3	48.5 45.7/54.6
2 weeks	115.5* 104.4/122.9	85.5 76.0/89.6	117.3* 108.0/128.5.3	95.0 81.4/108.3	54.0 47.7/59.9	47.0 38.9/50.3	54.0 47.0/56.6	69.3* 55.3/71.8
4 weeks	109.5 93.7/116.5	85.1 73.3/93.2	102.0 91.4/109.6	96.5 87.1/107.5	52.5 49.0/58.3	43.5 41.3/50.3	51.0 43.7/57.6	67.5* 95.3/117.5

*Notes*. *p < 0.05. The volunteers were screened, enrolled, and randomized into the four major groups of the study. Serum DHA and EPA concentrations were measured at the “0” time‐point as well as after the second and fourth weeks of intervention as described in the “Materials and Methods” Section.

EPA levels changed in a more conservative fashion. No increase in EPA concentration was seen at any time‐point of the study in volunteers ingesting DHA alone nor when DHA was ingested with lycopene as two separate formulations. However, serum EPA levels were significantly increased by 42.8% and 39.1% after the 2nd and 4th weeks of the study, respectively, in volunteers ingesting lycosome formulation of DHA containing 7 mg of lycopene (Table 2).

Table 3 shows the changes in serum lycopene level over the course of the study. As can be seen, ingestion of DHA alone did not affect serum lycopene concentration. However, 4‐week supplementation with lycopene itself resulted in an increase of its serum concentration by 47.3%. An almost similar increase took place in volunteers ingesting DHA and lycopene as two separate formulations. It is important to point out that lycosome formulated lycopene combined with DHA gave a more significant buildup of serum lycopene (*p* < 0.05) than lycopene alone, or the combined intake of DHA and lycopene ingested by volunteers as two separate formulations. In this case, the serum lycopene level was increased by nearly threefold over the value recorded for volunteers in the pretreatment period.

**Table 3:** Serum lycopene levels (medians with 95%/5% Cis)

Time point	Lycopene ng/mg of cholesterol			
	DHA	Lycopene	Co‐ingestion	Lycopene‐DHA
“0” week	163.0 159.0/172.9	168.0 160.4/175.0	172.5 158.7/178.5	172.0 159.7/1,816
4 weeks	169.5 162.5/177.9	247.5* 235.4/265.9	231.5* 220.3/239.6	481.0* 452.3/490.0

*Notes*. *p < 0.05. The volunteers were screened, enrolled, and randomized into the four major groups of the study. Serum lycopene concentrations were measured at the “0” time‐point as well as after the fourth week of intervention as described in the “Materials and Methods” section.

### 3.3 Lipid profile

Some important changes were observed in the serum lipid profile. There was a tendency towards reduction of serum triglyceride level in patients supplemented with DHA alone as well as for the group where DHA was taken with a separate lycopene capsule, reduction of medians by 6.0 and 9.5 mg/dl respectively. However, in patients who were given the DHA lycosome formulation, the reduction in triglycerides was significantly greater than in the two previous groups, by 27.5 mg/dl. Supplementation with lycopene alone did not affect concentration of these lipids (Table 4).

**Table 4:** Serum LDL and triglycerides levels (medians with 95%/5% Cis)

Time point	LDL mg/dl				Triglycerides mg/dl			
	DHA	Lycopene	Co‐Ingestion	Lycopene‐DHA	DHA	Lycopene	Co‐Ingestion	Lycopene‐DHA
“0” week	155.5 150.3/158.3	156.0 149.7/160.3	155.5 151.7/159.3	153 149.7/158.3	191.5 180.4/200.6	203.5 191.4/210.6	199.0 187.0/206.9	194.5 181.8/206.8
4 weeks	153.5 148.4/157.3	155.5 148.7/159.0	151.5 147.0/159.0	133.5* 123.1/138.2	185.5 180.4/200.9	190.0 181.8/199.3	189.5 185.7/199.9	167.0* 160.4/187.3

*Notes*. *p < 0.05. The volunteers were screened, enrolled, and randomized into the four major groups of the study. Serum low‐density lipoproteins (LDL) and triglyceride concentrations were measured at the “0” time‐point as well as after the fourth week of intervention as described in the “Materials and Methods” section.

An unexpected finding was a significant reduction in LDL cholesterol in the group which took the DHA lycosome formulation, by 19.5 mg/dL. This was accompanied by a reduction in the total cholesterol (results not shown). In the three other groups, there were no significant changes in either of these cholesterol parameters by the end of the study.

No changes in serum HDL level were observed in our study (results not shown).

### 3.4 Oxidative damage

Changes in the lipid profile were accompanied by a reduction in serum malonic dialdehyde level (Table 5). Although DHA ingested alone did not cause a statistically significant decrease in IOD values (*p* > 0.05), supplementation with lycopene taken as a singular formulation reduced IOD values by 19.8% (*p* < 0.05). This decline was more pronounced (by 43.1%) in volunteers who ingested DHA and lycopene as two separate formulations. However, lycosome formulation of DHA caused the most prominent reduction in serum IOD values (decrease by 51.9%, *p* < 0.05).

**Table 5:** Oxidized LDL and inflammatory oxidative damage (IOD) (medians with 95%/5% Cis)

Time point	Oxidized LDL				IOD malonic dialdehyde μM			
	DHA	Lycopene	Co‐Ingestion	Lycopene‐DHA	DHA	Lycopene	Co‐Ingestion	Lycopene‐DHA
“0” week	727.0 666.3/749.8	706.0 688.6/743.3	721.5 546.7/747.9	727.5 654.3/776.1	136.0 119.0/141.9	146.5 139.0/155.9	151.0 141.1/160.2	145.5 135.1/159.2
4 weeks	681.0 620.4/711.5	660.5 605.7/695.1	579.0* 433.9/610.8	427.0* 378.5/480.9	115.5 103.4/120.6	117.5* 101.0/122.9	86.0* 76.7/98.5	70.0* 50.1/75.9

*Notes*. *p < 0.05. The volunteers were screened, enrolled, and randomized into the four major groups of the study. Oxidised LDL and IOD malonic dialdehyde values were measured at the “0” time‐point as well as after the fourth week of intervention as described in the “Materials and Methods” section.

These changes found some reflection in oxidized LDL levels. Once again lycosome formulation of DHA caused the most significant reduction of oxidized LDL (reduction by 41.3% of control value). Co‐ingestion of DHA and lycopene as two separate formulations was less effective (decrease by 19.7%). A less substantial decrease in oxidized LDL values was seen in volunteers who ingested lycopene alone (reduction by 6.4%), whereas DHA alone did not cause any changes in oxidized LDL levels.

### 3.5 Tissue oxygenation

Table 6 demonstrates, that in all three groups of volunteers supplemented with lycopene whether in a separate capsule form or in the lycosome formulation with DHA, peripheral tissue oxygen saturation was significantly improved by the end of the study by 9.5% and 14.5%, respectively. In the group of DHA alone, StO_2_ was only slightly increased, by 6.1%. By the end‐point of the observational period, the values of plasma oxygen transport for all groups of patients were increased by a similar level of 11.0%–23.3% over the baseline level.

**Table 6:** Plasma oxygen transport and tissue oxygen saturation (medians with 95%/5% Cis)

Time point	Plasma oxygen transport				Tissue oxygen saturation			
	DHA	Lycopene	Co‐Ingestion	Lycopene‐DHA	DHA	Lycopene	Co‐Ingestion	Lycopene‐DHA
“0” week	634.0 618.0/650.8	648.5 639.4/652.6	640.3 623.7/657.5	639.5 630.1/651.1	73.0 69.3/82.9	75.0 72.3/77.6	75.5 71.0/77.6	76.5 73.0/77.6
4 weeks	638.5 610.8/693.0	786.0* 729.0/827.5	711.0 650.8/757.1	789.0* 771.3/829.1	77.5 75.0/82.9	79.0 75.3/81.0	85.0* 79.0/86.6	91.0* 86.4/92.3

*Notes*. *p < 0.05. The volunteers were screened, enrolled, and randomized into the four major groups of the study. Plasma oxygen transport and tissue oxygen saturation values were measured at the “0” time‐point as well as after the fourth week of intervention as described in the “Materials and Methods” section.

## 4 DISCUSSION

Recent consensus among medical professionals, health authorities, and dieticians suggests a recommended daily intake of DHA/EPA for the general population of 500 mg/day (Salem & Eggersdorfer, 2015). Higher doses of DHA/EPA either in the form of fish oil or algae extracts, or as synthetic formulations have been shown to be beneficial for cardiovascular health parameters including postprandial hypertriglyceridemia and HDL levels but are likely to be associated with some minor side effects (heartburn, nausea, diarrhea, rash, nosebleeds) and some major (pancreatitis, fibromyalgia, arthritis, arrhythmias, hypertension, kidney failure) (Bays, 2007; Lien, 2009). The possibility of increased levels of serum LDL represents another concern regarding the use of DHA (Asztalos et al., 2016) Therefore, strategies for DHA use for health purposes often translate into balancing act between increasing doses and emerging side effects. Consideration of the limited bioavailability of n‐3‐PUFA has become an essential factor in the development of new nutraceutical formulations allowing harmless and effective medicinal use of low dose DHA supplementation regimens.

The major conclusion from this study is that reducing the DHA dose not only without compromising but enhancing and expanding its health benefits is an achievable task when bioavailability limits of DHA are counteracted by implementation of lycosome technology. First of all, our results are consistent with multiple reports describing the limited bioavailability of DHA. As we have shown above, a month‐long daily ingestion of the regular formulation of DHA at 250 mg without lycopene does not translate into a significant increase in serum DHA and EPA levels. In particular, a 2‐week ingestion of regular DHA led only to a modest (24.8% increase) in DHA concentration over basal values with no statistically significant changes observed in serum DHA or EPA at the end‐point of the study. Interestingly, ingestion of the lycosome formulation of DHA containing 7 mg of lycopene was not accompanied by any significant changes in the serum level of DHA at any time‐point in the study. One of the possible explanations could be that lycopene, as a part of the lycosome formulation of DHA, may affect postabsorption traffic of the fatty acids and concentrate them in the liver. It is known that the tissues of this organ contain one of the highest concentrations of carotenoid receptors in the body. Therefore, the use of lycosomes containing such a powerful ligand for these receptors as lycopene could promote intrahepatic delivery of DHA and other essential fatty acids as “cargo” molecules (Petyaev, 2015).

Hepatocytes are the main cells responsible for the synthesis of triglycerides. Hence, by concentrating DHA, as an inhibitor of this synthesis, in the liver, it could be possible to maximize its metabolic effect on triglyceride biosynthesis. Those molecules of DHA which are present in the circulation, may be uptaken by other tissues expressing carotenoid receptors, and therefore, DHA blood level may not necessarily reflect DHA hepatic delivery.

As we have shown, there was a statistically significant reduction in the serum of not only triglycerides but also of LDL cholesterol in the hypercholesterolemic individuals supplemented with the lycosome‐formulated DHA containing 7 mg of lycopene. The LDL‐lowering DHA effect was not observed when conventional formulations of DHA or lycopene ingested together or separately were used. While the reduction in triglyceride values with DHA treatment is an anticipated phenomenon and widely acknowledged (Bowen et al., 2016; Maehre et al., 2015), the LDL reduction in individuals who ingested the lycosome formulation of DHA is a rather unexpected finding in our study which requires special consideration and further analysis. It is widely acknowledged that conventional DHA tends to increase LDL values in serum, whereas lycopene and EPA are well known to counteract increases in oxidized LDL and LDL‐cholesterol values (Petyaev, 2016a,2016b; Tajuddin, Shaikh, & Hassan, 2016). Detailed molecular analysis of the interplay between DHA and lycopene resulting in the reduction of LDL observed in our study might be related to EPA action and needs to be addressed in larger clinical settings, cultured cells, and possibly animal models.

Another projection comes from the analysis of oxidative status of volunteers enrolled in the study. As we have shown, supplementation with lycosome formulation of DHA containing 7 mg of lycopene resulted in the strongest, in comparison with three other groups, inhibition of malonic dialdehyde in the serum of volunteers in comparison with three other groups. Most importantly, this effect was accompanied by a reduction in oxidized LDL, which is a leading player in the initiation and development of atherosclerosis. It should be clarified that lycopene is the most powerful natural antioxidant whose anti‐radical capacity exceeds the antioxidant properties of all other carotenoids (Petyaev, 2016a,2016b). Therefore, increased bioavailability of lycopene and DHA which according to our results, seems to have a synergistic effect on the malonic dialdehyde level translates into a reduction in the major metabolic risk factor for atherosclerosis in hypercholesterolemic individuals treated with the lycosome formulation of DHA containing lycopene.

Another intriguing set of data was obtained when the parameters reflecting buildup of subclinical tissue hypoxia in hyperlipidemic patients were analyzed. As reported above, the lycosome formulation of DHA containing lycopene upregulated both the plasma oxygen transport and tissue oxygen saturation parameters. To the best of our knowledge, the effects of DHA and lycopene on the oxygen delivery system as well as tissue oxygen saturation have not been reported previously and unveil a possible new strategy in the correction of biological respiration for individuals with risk factors for CVD. In our opinion, upregulation of oxygen balance in tissues might be related to the previously described mitochondria‐stabilizing effect of lycopene (Petyaev, 2016a,2016b; Yi, He, & Wang, 2013) as well as the well‐known anti‐inflammatory action of DHA which in turn normalizes microcirculation (Bowen et al., 2016).

On the other hand, our study has some limitations. First of all, the intimate mechanisms behind the effect of lycosome formulation of DHA containing lycopene on lipid metabolism and oxygen homeostasis need to be thoroughly investigated in different clinical and experimental settings on a larger scale. Longer duration of treatment with lycosome‐formulated DHA should be tested in future work. DHA and EPA are known to improve HDL levels in volunteers and activate mechanisms of reverse cholesterol transport as reported by many authors (Baker, Miles, Burdge, Yaqoob, & Calder, 2016). Therefore, prolonged supplementation with lycosome‐formulated DHA may be effective in the correction of HDL values and development of new strategies for the correction of abnormalities of lipid homeostasis in atherosclerosis and CVD.

## CONFLICT OF INTEREST

Lycotec Ltd developed DHA‐Lycosome, which was evaluated in this study. Ivan M. Petyaev and Nigel H. Kyle are employees of Lycotec; Pavel Y. Dovgalevsky, Natalya E. Chalyk and Victor A. Klochkov are employees of the Institute of Cardiology in Saratov, Russian Federation. There have never been any financial relationships between Lycotec and the Cardiology Institute.

## ETHICAL STATEMENTS

The study protocol was evaluated and approved by the Institutional Review Boards of the Saratov Institute of Cardiology (Russian Federation) and Lycotec Ltd (UK). All study participants were informed about the study goals and signed consent forms were obtained from all volunteers. The study was conducted in accordance with the Declaration of Helsinki and the Guidelines accepted by USA and European agencies for clinical studies as well as current regulatory requirements for medical research and clinical trials in the Russian Federation.
